# Efficiency of high cumulative cisplatin dose in high‐ and low‐risk patients with locoregionally advanced nasopharyngeal carcinoma

**DOI:** 10.1002/cam4.4477

**Published:** 2021-12-03

**Authors:** Yu‐Ting Jiang, Kai‐Hua Chen, Jie Yang, Zhong‐Guo Liang, Ling Li, Song Qu, Xiao‐Dong Zhu

**Affiliations:** ^1^ Department of Radiation Oncology, Guangxi Medical University Cancer Hospital Nanning Guangxi China; ^2^ Department of Oncology, Affiliated Wuming Hospital of Guangxi Medical University Nanning Guangxi China

**Keywords:** concurrent chemotherapy, cumulative cisplatin dose, induction chemotherapy, nasopharyngeal carcinoma, nomogram

## Abstract

**Background:**

The optimal cumulative cisplatin dose (CCD) during radiation therapy for locoregionally advanced nasopharyngeal carcinoma (LA‐NPC) patients receiving induction chemotherapy (IC) plus CCRT remains controversial. This study aimed to explore the treatment efficiency of CCD for high‐and low‐risk patients with LA‐NPC.

**Methods:**

Data from 472 LA‐NPC patients diagnosed from 2014 to 2018 and treated with IC plus CCRT were reviewed. After propensity score matching, the therapeutic effects of a CCD > 200 and CCD ≤ 200 mg/m^2^ were evaluated comparatively. Five factors selected by multivariate analysis were incorporated to develop a nomogram. Subgroup analysis was conducted to explore the role of different CCDs in nomogram‐defined high‐ and low‐risk groups. Additionally, acute toxicities were evaluated comparatively between the high‐ and low‐CCD groups.

**Results:**

After matching, there was no difference between different CCD groups for all patients in terms of 3‐year overall survival (OS), distant metastasis‐free survival (DMFS), locoregional recurrence‐free survival (LRRFS), or progression‐free survival (PFS). A nomogram was built by integrating pretreatment EBV DNA, clinical stage, and post‐IC EBV DNA, post‐IC primary gross tumor and lymph node volumes obtained a C‐index of 0.674. The high‐risk group determined by the nomogram had poorer 3‐year PFS, OS, DMFS, and LRRFS than the low‐risk group. A total of CCD > 200 mg/m^2^ increased the survival rates of 3‐year PFS and DMFS (PFS: 72.5% vs. 54.4%, *p* = 0.012; DMFS: 81.9% vs. 61.5%, *p* = 0.014) in the high‐risk group but not in the low‐risk group. Moreover, the high CCD increased treatment‐related acute toxicities.

**Conclusions:**

A high CCD was associated with better 3‐year PFS and DMFS rates than a low dose for high‐risk patients but could not produce a survival benefit for low‐risk patients.

## Graphical abstract

This study compared the therapeutic effects of a cumulative cisplatin dose (CCD) > 200 and CCD ≤ 200 mg/m^2^ for patients with locoregionally‐advanced nasopharyngeal carcinoma receiving induction chemotherapy plus concurrent chemoradiotherapy. Based on a prognostic model, we found a high CCD was associated with better 3‐year progression‐free survival and distant metastasis‐free survival rates than a low dose for high‐risk patients but could not produce a survival benefit for low‐risk patients.

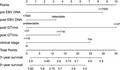

## 1 INTRODUCTION

Nasopharyngeal carcinoma (NPC) is a specific malignancy that is rare worldwide but frequently diagnosed in southern China.^1^, ^2^ Radiation therapy is recognized as the mainstay treatment for non‐metastatic NPC considering its deep‐seated anatomical location and high sensitivity to radiation.^3^ Among them, most patients are staged as locoregionally advanced disease (LA‐NPC), and concurrent chemoradiation (CCRT) has been recommended as the standard regimen.^4^, ^5^, ^6^ It has been widely proven that the addition of induction chemotherapy (IC) before CCRT is superior to CCRT alone for LA‐NPC patients due to its high efficacy for improving long‐term survival,^7^, ^8^, ^9^, ^10^, ^11^, ^12^, ^13^, ^14^ establishing this treatment as one of the most recommended care strategies for LA‐NPC. However, approximately 20% of patients still experience treatment failure.^15^ Under this situation, the question of identifying patients at different risk levels of disease progression and guiding individualized treatment is worth exploring.

Cisplatin‐based concurrent chemotherapy during CCRT is closely associated with a survival benefit.^4^, ^6^ The standard concurrent cisplatin protocols include weekly regime (30–40 mg/m^2^) and 3‐weekly regime (100 mg/m^2^).^5^, ^16^, ^17^ A cumulative cisplatin dose (CCD) during radiation therapy of 200 mg/m^2^ was regarded as the optimal dosage in the CCRT era without IC.^18^ However, the optimal CCD remains controversial for LA‐NPC treated with both IC and CCRT.

In this study, we evaluated the role of CCD in LA‐NPC patients receiving IC. Furthermore, a subgroup analysis was performed to investigate the treatment efficiency of CCD for patients in the high‐ and low‐risk groups based on a nomogram. Our findings will help guide the modification of the intensity of CCRT.

## 2 MATERIALS AND METHODS

### 2.1 Patients

Between 2014 and 2018, a total of 427 consecutive patients in our center were screened for this study if they met the following criteria: (1) newly histologically diagnosed with stage III‐IVa NPC (restaged in accordance with AJCC/UICC 8th edition); (2) treated using a combination of 2 to 4 cycles of IC and single‐agent cisplatin‐based CCRT; (3) without additional adjuvant chemotherapy, targeted therapy or immunotherapy; (4) no history of antitumor treatment before our study; (5) available clinical information, examination, and follow‐up data; and (6) no serious diseases or secondary malignancy when diagnosed with NPC. The study was approved by the Medical Ethics Committee of Guangxi Medical University Cancer Hospital. Before treatment, informed consent was acquired from all patients.

### 2.2 Treatment and evaluation

All treatments were performed according to the treatment protocol of our institution. IC regimens included the TPF, TP, PF, and GP regimes, which were conducted every three weeks for two to four cycles before CCRT. Concurrent chemotherapy was triple‐weekly cisplatin regimen with a dose of 80–100 mg/m^2^ for one to three cycles. Treatment‐related acute toxicities were classified by the Common Toxicity Criteria for Adverse Events version 4.0 (CTCAE 4.0).

All patients underwent radical IMRT. The radiation doses of primary gross tumor volume (GTVnx), cervical lymph node tumor volume (GTVnd), high‐risk clinical target volume (CTV1), and low‐risk clinical target volume (CTV2) were 70.6–72.6 Gy/31–32 f, 60.0–72.3 Gy/30–32 f, 60–64 Gy/30–32 f, and 54–55.8 Gy/30–32 f, respectively.

The cutoff value for the EBV DNA level after IC (post‐EBV DNA) was defined as detectable/undetectable (1000 copies/ml), whereas the cutoff value for pretreatment EBV DNA level (pre‐EBV DNA), post‐IC primary gross tumor (post‐GTVnx), and lymph node (post‐GTVnd) volumes were set at 7000 copies/ml, 118 and 37 cm^3^, respectively, based on receiver operator characteristic (ROC) curve analyses.

Detailed information on chemotherapy, radiotherapy, measurements of EBV DNA level and tumor volumes was reported in our previous study^19^ and is also shown in the Supporting Information materials.

### 2.3 Follow‐up

Patients received an outpatient examination or telephone interview follow‐up after treatment. All patients were regularly screened via physical examination, nasopharyngoscopy, and imaging every 3 months for the first 2 years after radiotherapy, 6 months for the next 3 years, and annually thereafter until death. Progression‐free survival (PFS) was the main endpoint. The secondary endpoints included overall survival (OS), distant metastasis‐free survival (DMFS), and locoregional relapse‐free survival (LRRFS). PFS was defined as the date from the initiation of histological diagnosis to first disease progression, death, or last follow‐up. OS, DMFS, and LRRFS were defined as the date from initiation of histological diagnosis to death, first distant metastasis, and first locoregional relapse, respectively, or last follow‐up.

### 2.4 Nomogram development and validation

We performed multivariate Cox regression analyses using backward stepwise selection to select the independent predictors, which were incorporated to generate a nomogram. The predictive performance of the nomogram was evaluated for discrimination and calibration ability. Discrimination was measured via Harrell's concordance index (C‐index), which was calculated for 3‐ and 5‐year PFS rates by 1000 bootstrap resamples.^20^ The discriminative ability of the nomogram was determined by time‐dependent receiver operating characteristic (tdROC) curve analysis.^21^ Calibration was evaluated using the calibration curve to compare the observed PFS with predicted survival. Based on the total score calculated by the nomogram, patients were separated into two risk groups for therapeutic value assessment of different CCDs.

### 2.5 Statistical analysis

We used SPSS (version 25.0) and R software (version 3.6.3) to complete statistical analyses. Categorical variables (sex, pathological type, tumor stage, IC regimen, post‐EBV DNA, and IC cycles) were classified based on clinical knowledge, and numerical variables (age, pre‐EBV DNA, post‐GTVnx, and post‐GTVnd) were converted to categorical variables according to the cutoff values determined by ROC curve analyses. Categorical variables are presented as whole numbers and proportions. The Pearson *X* ^2^ test or Fisher's exact test was used to evaluate the differences in proportions of patients' baseline characteristics and acute toxicity between the CCD ≤ 200 and CCD > 200 mg/m^2^ groups. To minimize the influence of selection bias by potential confounding factors, 1:1 propensity score matching (PSM) was conducted to compare baseline clinicopathological characteristics between the two groups with the nearest neighbor‐matching method and a caliper of 0.05 (by the package “Matchlt” in R). Kaplan–Meier analysis and the log‐rank test were performed to calculate survival rates and compare the differences (by the package of “survival” in R).

The nomogram included all independent predictors that were identified by univariable and multivariable analyses (by the package “rms” in R). Finally, according to the cutoff value of the risk score calculated by a ROC curve, the whole cohort was classified into high‐ and low‐risk groups.

Two‐sided *p* < 0.05 was regarded as statistically significant.

## 3 RESULTS

### 3.1 Patient characteristics and survival outcomes

A total of 427 stage III‐IVa patients were recruited for this study. Of these, 305 patients who received a CCD ≤ 200 mg/m^2^ and 122 patients were treated with a CCD > 200 mg/m^2^. The baseline characteristics of patients grouped by different CCDs before and after matching are summarized in Table 1. In the original cohort, the outcomes indicated a significantly greater percentage of male (*p* = 0.002) and younger patients (*p* = 0.01) received a high CCD. Post‐GTVnx (*p* = 0.031) and post‐GTVnd (*p* = 0.037) were also significantly associated with CCD. There was no significant association between other clinicopathological features and CCD. After one‐to‐one PSM was conducted, a total of 111 pairs were selected for further analysis. The median age was 43 years (range, 16−72), 191/222 (86.0%) patients were male and 31/222 (14.0%) patients were female. 87/222 (39.2%) patients were categorized as stage III NPC and 135/222 (60.8%) were categorized as stage IVa NPC. Baseline characteristics were well balanced between the two arms with all P values exceeding 0.169.

**TABLE 1:** Baseline characteristics of the entire cohort before and after matching

Characteristics	Before matching			After matching		
	CCD ≤ 200 (*n* = 305)	CCD > 200 (*n* = 122)	*p*‐value	CCD ≤ 200 (*n* = 111)	CCD > 200 (*n* = 111)	*p*‐value
Age (years)			0.010			0.881
Median (range)	17–73	16–67		17–72	16–67	
<50	182 (59.7)	89 (73.0)		81 (73.0)	79 (71.2)	
≥50	123 (40.3)	33 (27.0)		30 (27.0)	32 (28.8)	
Sex			0.002			0.699
Female	91 (29.8)	19 (15.6)		14 (12.6)	17 (15.3)	
Male	214 (70.2)	103 (84.4)		97 (87.4)	94 (84.7)	
Pathological type			0.472			0.822
WHO type I/II	32 (10.5)	10 (8.2)		12 (10.8)	10 (9.0)	
WHO type III	273 (89.5)	112 (91.8)		99 (89.2)	101 (91.0)	
T stage			0.449			0.656
T1	6(2.0)	2 (1.6)		1 (0.9)	2 (1.8)	
T2	82 (26.9)	24 (19.7)		29 (26.1)	22 (19.8)	
T3	108 (35.4)	46 (37.7)		38 (34.2)	43 (38.7)	
T4	109 (35.7)	50 (41.0)		43 (38.7)	44 (39.6)	
N stage			0.553			0.970
N0	5 (1.6)	1(0.8)		1 (0.9)	1 (0.9)	
N1	90 (29.5)	42 (34.4)		35 (31.5)	35 (31.5)	
N2	118 (38.7)	49 (40.2)		42 (37.8)	45 (40.5)	
N3	92 (30.2)	30 (24.6)		33 (29.7)	30 (27.0)	
Clinical stage			0.925			1.000
III	124 (40.7)	49 (40.2)		43 (38.7)	44 (39.6)	
IVa	181 (59.3)	73 (59.8)		68 (61.3)	67 (60.4)	
Pre‐EBV DNA (copies/ml)			0.699			0.304
<7000	199 (65.2)	82 (67.2)		82 (73.9)	74 (66.7)	
≥7000	106 (34.8)	40 (32.8)		29 (26.1)	37 (33.3)	
Post‐EBV DNA (copies/ml)			0.499			0.764
Undetectable	220 (72.1)	84 (68.9)		82 (73.9)	79 (71.2)	
Detectable	85 (27.9)	38 (31.2)		29 (26.1)	32 (28.8)	
post‐GTVnx (cm^3^)			0.031			0.652
<118	238 (78.0)	83 (68.0)		82 (73.9)	79 (71.2)	
≥118	67 (22.0)	39 (32.0)		29 (26.1)	32 (28.8)	
post‐GTVnd (cm^3^)			0.037			0.893
<37	164 (53.8)	52 (42.6)		49 (44.1)	50 (45.9)	
≥37	141 (46.2)	70 (57.4)		62 (55.9)	60 (54.1)	
IC regimen			0.187			0.169
TPF	263 (86.2)	99 (81.1)		100 (90.1)	92 (82.9)	
TP/PF/GP	42 (13.8)	23 (18.9)		11 (9.9)	19 (17.1)	
IC cycles			0.968			1.000
2	53 (17.4)	21 (17.2)		18 (16.2)	17 (15.3)	
3/4	252 (82.6)	101 (82.8)		93 (83.8)	94 (84.7)	

Abbreviations: CCD, cumulative cisplatin dose; EBV, Epstein–Barr virus; GP, cisplatin and gemcitabine; IC, induction chemotherapy; PF, cisplatin and 5‐fluorouracil; post‐EBV DNA, post‐IC EBV DNA; post‐GTVnd, post‐IC cervical lymph node tumor volume; post‐GTVnx, post‐IC primary gross tumor volume; pre‐EBV DNA, pretreatment EBV DNA; TP, docetaxel and cisplatin; TPF, docetaxel, cisplatin, and 5‐fluorouracil; WHO, World Health Organization.

The median follow‐up duration of the whole cohort was 46 months (range, 5−89 months). Upon the last follow‐up, 92 patients (21.5%) died, 80 (18.7%) experienced distant metastasis, 35 (8.2%) developed locoregional recurrence, and 130 (30.4%) developed disease progression. The 3‐year OS, DMFS, LRRFS, and PFS were 86.2%, 83.6%, 93.2%, 76.5%, respectively, and the 5‐year rates were 74.1%, 78.4%, 90.3%, 65.0%, respectively.

### 3.2 Relationship between CCD and clinical outcome

**FIGURE 1:**
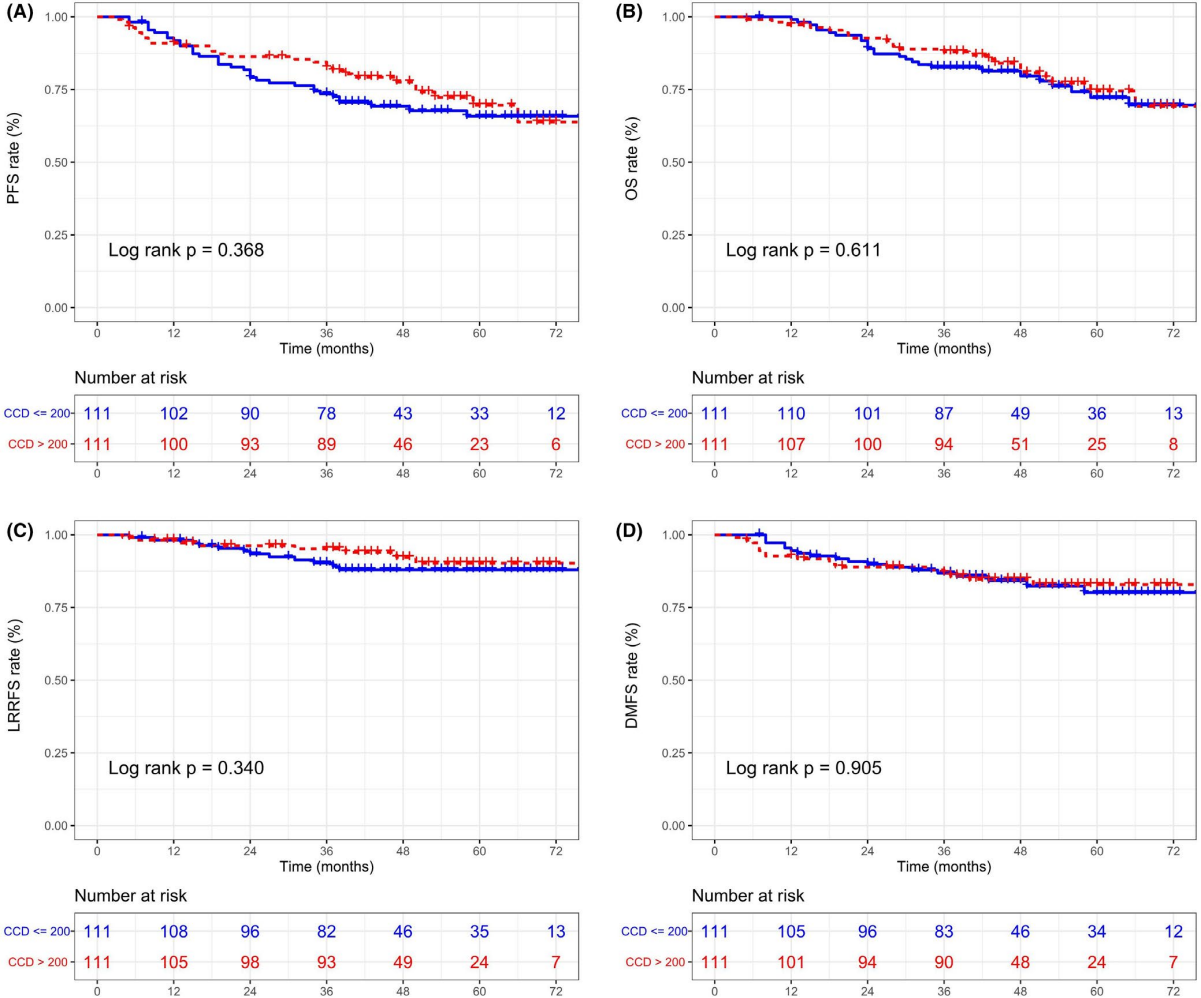
Progression‐free survival (PFS) (A), overall survival (OS) (B), locoregional recurrence‐free survival (LRRFS) (C), and distant metastasis‐free survival (DMFS) (D) Kaplan–Meier curves between cumulative cisplatin dose (CCD) ≤ 200 mg/m^2^ and CCD > 200 mg/m^2^ groups within 222 nasopharyngeal carcinoma patients after matching

After 1:1 nearest neighbor matching, 111 pairs were selected for the CCD ≤ 200 and CCD > 200 mg/m^2^ groups, with balanced baseline characteristics (all *p* > 0.05) (Table 1 **)**. We explored whether patients would benefit from a high CCD after PSM. No statistically significant differences were detected in the survival of patients in the two groups (all *p* > 0.05). Figure 1 depicts the survival curves of the matched groups. Additionally, the univariate analysis in the unmatched cohort indicated that CCD was not a potential prognostic factor (Table S1).

### 3.3 Prognostic factors

We performed univariate analysis of the candidate variables that might be predictors based on basic clinical knowledge and prognostic factors reported in previous studies. The concrete results of the above step are shown in Table S1. According to univariate analysis, the variables associated with lower survival rates were sex (male), advanced age (≥50 years), N stage, clinical stage, higher pre‐EBV DNA (≥7000 copies/ml), detectable post‐EBV DNA, and larger post‐GTVnx (≥118 cm^3^), post‐GTVnd (≥37 cm^3^) (all *p* < 0.05). The variables included in multivariate analyses were prognostic factors identified by univariate analysis. The visual details of this step for OS, DMFS, LRRFS, and PFS are shown in Table 2. Spearman correlation analysis suggested that pre‐EBV DNA has relationship with post‐EBV DNA, with a correlation coefficient of 0.523 (*p* < 0.001). Thus, post‐EBV DNA was not included in the multivariate analysis in Table 2, and another multivariate analysis combining post‐EBV DNA and other prognostic factors in addition to pre‐EBV DNA is shown in Table S2. According to multivariate analysis, pre‐EBV DNA, post‐EBV DNA, post‐GTVnx, post‐GTVnd, and clinical stage remained independent predictors.

**TABLE 2:** Multivariable Cox regression analysis in the entire cohort

Characteristics	HR (95% CI)	*p*‐value
Overall survival		
Age (years) (≥50 vs. <50)	1.404 (0.923–2.135)	0.112
Sex (male vs. female)	1.502 (0.872–2.587)	0.142
Clinical stage (IVa vs. III)	1.968 (1.180–3.283)	0.009
Post‐GTVnx (≥118 vs. <118)	1.594 (1.028–2.472)	0.037
Post‐GTVnd (≥37 vs. <37)	2.318 (1.490–3.607)	<0.001
Pre‐EBV DNA (≥7000 vs. <7000)	2.035 (1.336–3.100)	0.001
Progression‐free survival		
Clinical stage (IVa vs. III)	1.645 (1.101–2.457)	0.015
Post‐GTVnx (≥118 vs. <118)	1.309 (0.897–1.908)	0.162
Post‐GTVnd (≥37 vs. <37)	1.975 (1.379–2.829)	<0.001
Pre‐EBV DNA (≥7000 vs. <7000)	1.966 (1.384–2.791)	<0.001
Distant metastasis‐free survival		
Clinical stage (IVa vs. III)	1.744 (1.179–2.581)	0.005
Post‐GTVnd (≥37 vs. <37)	1.973 (1.377–2.826)	<0.001
Pre‐EBV DNA (≥7000 vs. <7000)	2.008 (1.417–2.846)	<0.001

Note

A Cox proportional hazards regression model was used to detect variables individually without adjustment. All variables were transformed into categorical variables. HRs were calculated for age (years) (≥50 vs. <50), sex (male vs. female), clinical stage (IVa vs. III), pre‐EBV DNA (≥7000 vs. <7000), post‐GTVnx (≥118 cm^3^ vs. <118 cm^3^), post‐GTVnx (≥37 cm^3^ vs. <37 cm^3^).

Abbreviations: CI, confidence interval; HR, hazard ratio. EBV, Epstein–Barr virus; post‐GTVnd, post‐IC cervical lymph node tumor volume; post‐GTVnx, post‐IC primary gross tumor volume; pre‐EBV DNA, pretreatment EBV DNA.

### 3.4 Establishment and evaluation of nomogram for PFS

**FIGURE 2:**
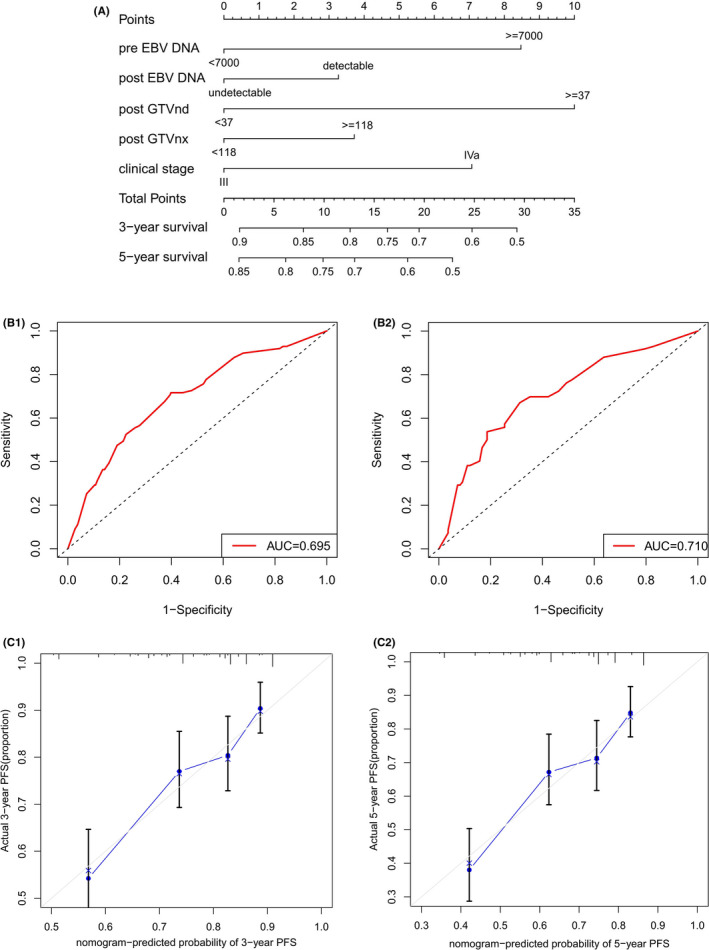
(A) Nomogram for 3‐ and 5‐year progression‐free survival (PFS) in all patients; (B) Nomogram's td‐receiver operator characteristic (td‐ROC) curves for 3‐ and 5‐year PFS; and (C) Nomogram's calibration curves for 3‐ and 5‐year PFS

According to the results of multivariable analyses, independent prognostic factors (pre‐EBV DNA, post‐EBV DNA, post‐GTVnx, post‐GTVnd, and clinical stage) were integrated into a nomogram for PFS (C‐index, 0.674; 95% CI, 0.627−0.721) (Figure 2A). Furthermore, td‐ROC analysis demonstrated good discriminatory ability (Figure 2B). Calibration curves suggested a good association between the nomogram predicted and actual observed probabilities (Figure 2C).

### 3.5 Risk stratification

Given the nomogram had good predictive ability, we used the nomogram to conduct risk stratification to determine the high‐risk group (total points > 19) and the low‐risk group (total points < 19) by a ROC curve. A significantly greater PFS was observed in patients with low risk (3‐year PFS rate: 83.8% vs. 55.4%; *p* < 0.001). Similarly, this trend was discovered in other 3‐year survival rates (OS: 93.0% vs. 66.9%; *p* < 0.001; DMFS: 89.7% vs. 67.6%; *p* < 0.001; LRFS: 94.8% vs. 88.6%; *p* = 0.050; Figure [Link], [Link], [Link], [Link]).

### 3.6 Subgroup analysis for the whole cohort based on nomogram

**FIGURE 3:**
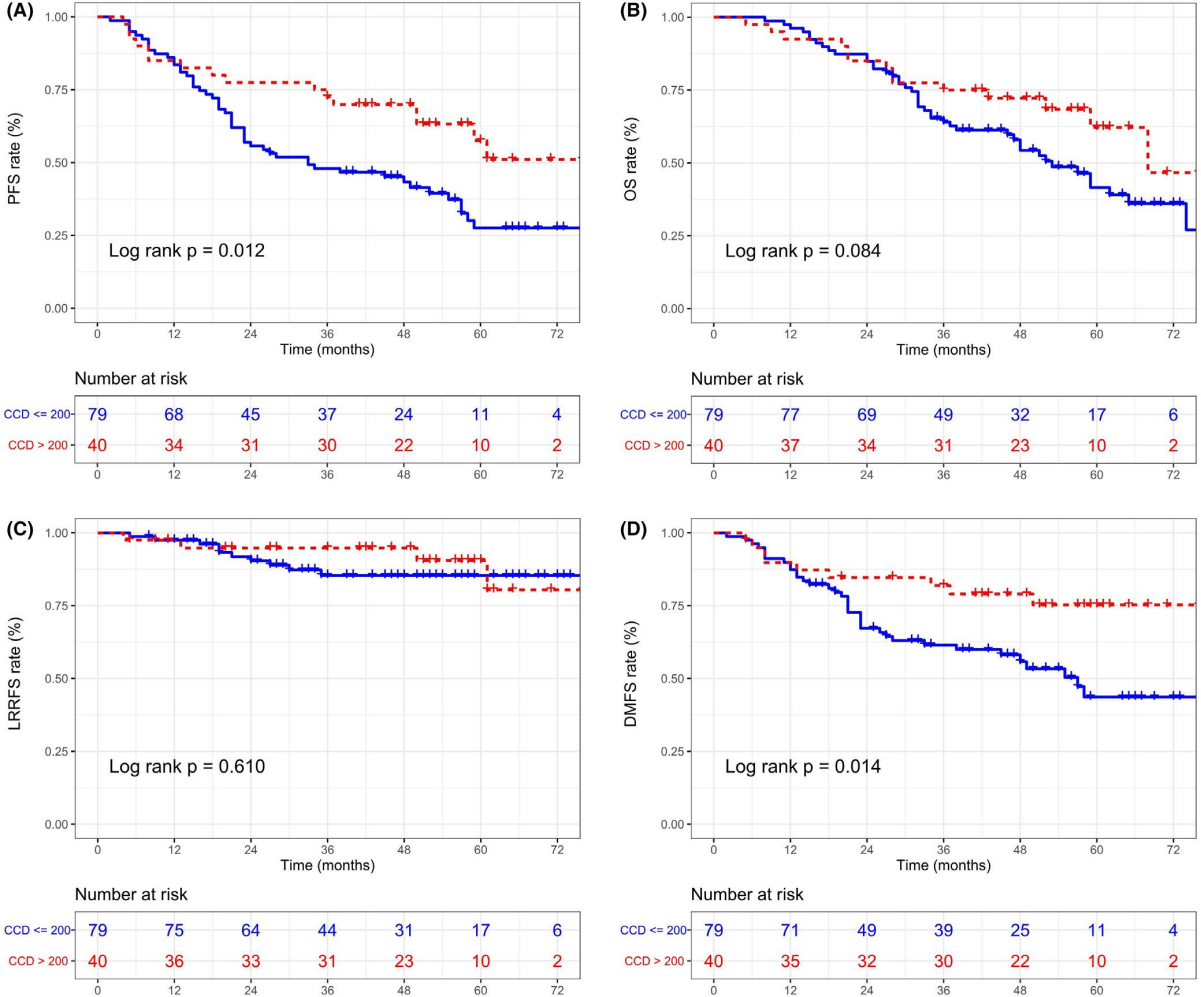
Progression‐free survival (PFS) (A), overall survival (OS) (B), locoregional recurrence‐free survival (LRRFS) (C), and distant metastasis‐free survival (DMFS) (D) Kaplan–Meier curves between cumulative cisplatin dose (CCD) < 200 mg/m^2^ and CCD ≥ 200 mg/m^2^ groups within nomogram‐defined high‐risk patients

**FIGURE 4:**
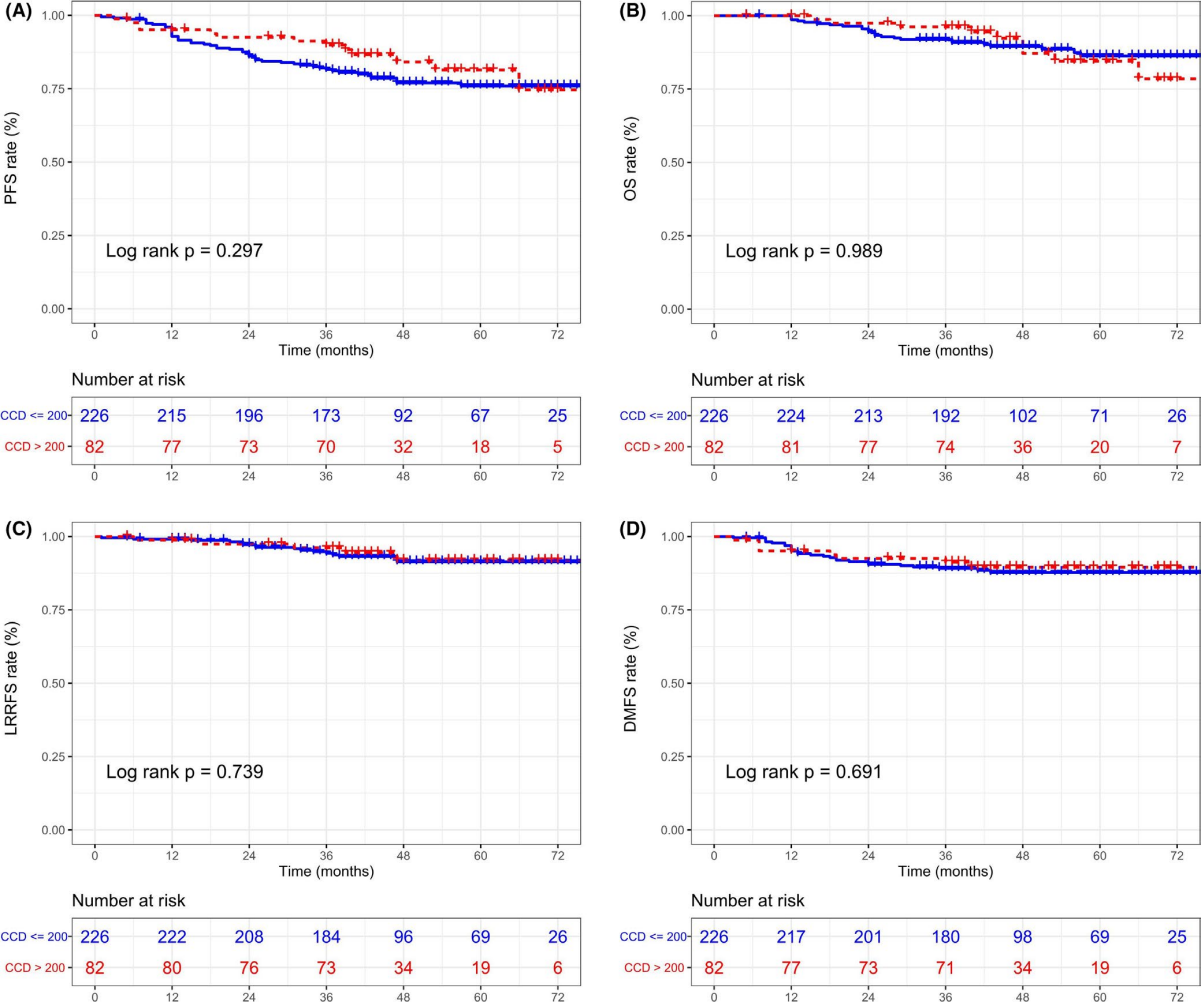
Progression‐free survival (PFS) (A), overall survival (OS) (B), locoregional recurrence‐free survival (LRRFS) (C), and distant metastasis‐free survival (DMFS) (D) Kaplan–Meier curves between cumulative cisplatin dose (CCD) < 200 mg/m^2^ and CCD ≥ 200 mg/m^2^ groups within nomogram‐defined low‐risk patients

Given that patients in different risk subgroups had different rates of disease progression, we comparatively evaluated the therapeutic efficacy of high and low CCDs in different risk subgroups. For patients with high risk, a total of CCD > 200 mg/m^2^ increased the 3‐year PFS and DMFS rates compared with a CCD ≤ 200 mg/m^2^ (PFS: 72.5% vs. 54.4%, *p* = 0.012; DMFS: 81.9% vs. 61.5%, *p* = 0.014) but not OS or LRRFS (OS: 75.0% vs. 64.0%, *p* = 0.084; LRRFS: 94.8% vs. 85.3%, *p* = 0.610) (Figure 3). However, in the low‐risk group, the application of different CCDs did not produce different therapeutic effects on 3‐year PFS (89.9% vs. 81.6%; *p* = 0.297), OS (96.2% vs. 91.9%; *p* = 0.989), DMFS (91.2% vs. 89.1%; *p* = 0.691), or LRRFS (96.2% vs. 94.3%; *p* = 0.739) (Figure 4).

### 3.7 Acute toxicity

During the CCRT period, we evaluated the treatment‐related acute toxicity between the CCD > 200 and CCD ≤ 200 mg/m^2^ groups. Details of the results are presented in Table 3. A high CCD significantly increased the incidences of grade 1–4 acute toxicities compared with a low CCD, such as gastrointestinal reactions (94.3% vs. 74.5%, *p* < 0.001) and hepatoxicity (ALT increase: 34.4% vs. 22.3%, *p* = 0.014; bilirubin increase, 34.4% vs. 8.5%, *p* < 0.001). However, intergroup differences in AST increase (25.4% vs. 17.7%) and hematological toxicities such as leukocytopenia (96.7% vs. 91.1%), neutropenia (75.4% vs. 77.7%), anemia (71.3% vs. 72.8%), and thrombocytopenia (16.4% vs. 22.0%) were not significant (all *p* > 0.05).

**TABLE 3:** Grade 1–4 acute toxicities during CCRT between the two different CCD groups

Variable	CCD ≤ 200 (*n* = 305)	CCD > 200 (*n* = 122)	*p*‐value
Leukocytopenia			
All	278 (91.1)	118 (96.7)	0.061
Grade 3/4	95 (31.1)	28 (23.0)	0.091
Neutropenia			
All	237 (77.7)	92 (75.4)	0.612
Grade 3/4	63 (20.7)	20 (16.4)	0.346
Anemia			
All	222 (72.8)	87 (71.3)	0.811
Grade 3/4	30 (9.8)	8 (6.6)	0.282
Thrombocytopenia			
All	67 (22.0)	20 (16.4)	0.196
Grade 3/4	10 (3.3)	2 (1.6)	0.420
AST increase			
All	54 (17.7)	31 (25.4)	0.081
Grade 3/4	0 (0)	0 (0)	1.000
ALT increase			
All	68 (22.3)	42 (34.4)	0.014
Grade 3/4	1 (0.3)	1 (0.8)	0.490
Bilirubin increase			
All	26 (8.5)	42 (34.4)	< 0.001
Grade 3/4	0 (0)	4 (3.2)	0.006
Gastrointestinal reactions			
All	221 (72.5)	115 (94.3)	< 0.001
Grade ¾	23 (7.5)	9 (7.4)	1.000

Abbreviations: ALT, alanine aminotransferase; AST, aspartate aminotransferase; CCD, cumulative cisplatin dose.

The results of the comparison of grade 3–4 adverse reactions showed that a CCD > 200 mg/m^2^ was associated with a greater bilirubin increase (3.2% vs. 0%; *p* < 0.006) However, incidences of other grade 3–4 toxicities were comparable between patients receiving high and low CCDs.

## 4 DISCUSSION

We undertook this study to explore the efficiency of a CCD > 200 mg/m^2^ in different risk patients with LA‐NPC treated with IC plus CCRT based on a nomogram. Our findings showed that for the whole LA‐NPC cohort, all survival endpoints did not achieve significant differences between the high and low CCD groups. The nomogram in our study incorporating pretreatment (clinical stage and pre‐EBV DNA) and post‐IC (post‐EBV DNA, GTVnx, and GTVnd) risk factors showed satisfactory value in risk stratification and has potential for guiding individualized decision‐making of CCD. A CCD > 200 mg/m^2^ improved the PFS and DMFS of patients in high‐risk subgroup identified by the nomogram but fail to bring significant benefit for patients with low risk. In clinical practice, it is crucial to identify subgroups that may benefit from a high CCD because IC may reduce patients' tolerance to the following CCRT.

Regarding the administration of a triple‐weekly cisplatin‐based concurrent chemotherapy regimen, physicians in most randomized trials and clinical practice usually prefer three cycles of chemotherapy for LA‐NPC patients, which makes the CCD over 200 mg/m^2^. However, a substantial proportion of patients failed to complete three cycles of chemotherapy due to toxicity and treatment costs. Additionally, there is no consensus that a CCD > 200 mg/m^2^ produces the greatest survival benefit for NPC patients treated with IC. In the current study, we first investigated whether all patients would benefit from an increasing CCD. After PSM, we found that a CCD > 200 mg/m^2^ showed similar therapeutic value as a CCD ≤ 200 mg/m^2^. Although a high CCD failed to significantly improve survival for all LA‐NPC patients, further subgroup analyses revealed that high‐risk patients defined by the nomogram acquired survival benefit from a CCD > 200 mg/m^2^ for 3‐year PFS and DMFS rates, while patients with low risk did not benefit. We also compared the acute toxicities of the different CCD groups during the period of CCRT. The most common acute toxicities included hematologic toxicities and gastrointestinal reactions. Our study also showed that the high CCD group could increase the incidences of grade 1–4 acute toxicities. If patients experience too many adverse events during CCRT, their physical status or fear of acute toxicities may decrease their tolerance to subsequent radical radiotherapy, which may have an adverse effect on survival. According to our data, routine practice of three cycles of triple‐weekly concurrent cisplatin for “all” LA‐NPC patients who have received IC should be reconsidered.

Throughout the long history of treatment, several studies have explored the optimal CCD for LA‐NPC patients. Wei et al. indicated that a CCD > 200 mg/m^2^ was associated with better PFS for stage II‐IVa NPC patients.^22^ However, patients enrolled in this study received only CCRT without IC. In accordance with some results of our study, another study indicated that for LA‐NPC patients treated with additional IC, a CCD > 200 mg/m^2^ failed to significantly improve the prognosis for 5‐year OS, PFS, DMFS and LRFS.^23^ In addition, Liu et al. demonstrated that patients with CR/PR could benefit from a CCD ≥ 200 mg/m^2^ for DMFS and PFS, but those with SD/PD could not, and they also demonstrated that the higher CCD was significantly associated with more acute toxicities.^24^ Wen et al. indicated that subgroups with high risk (higher pretreatment EBV DNA level or advanced stage) who received a CCD ≥ 200 mg/m^2^ have better 5‐year PFS, OS, and DMFS, but there was no difference in the low‐risk group between high and low CCDs.^25^ Several studies have also suggested that post‐IC EBV DNA level and IC cycles would be useful factors for assessing the treatment efficiency of CCD.^23^, ^26^ The purpose of CC is to yield effective antitumor effects based on acceptable toxicity. With the widespread use of IC in patients with LA‐NPC, a significant number of patients cannot tolerate a CCD > 200 mg/m^2^ after IC. Thus, finding a more suitable CCD for patients with different risks is necessary, which may not only confer a survival advantage but also decrease the economic cost and occurrence of toxicities.

Pretreatment predictors include some biomarkers and initial clinical stages, may not be sufficient to predict the prognosis because the primary tumors and metastatic lymph nodes usually change after IC. Performing IC prior to CCRT provides a unique opportunity to assess the chemotherapy sensitivity of tumors, which may contribute to risk stratification and individualized treatment. In our results, five pretreatment and post‐IC characteristics (clinical stage, pre‐EBV DNA, post‐EBV DNA, GTVnx, and GTVnd) were proven to be independent factors after multivariate analyses. Some studies have proved that the above five factors have potential value in predicting prognosis of NPC.^23^, ^25^, ^26^, ^27^, ^28^ It seems reasonable that patients at high risk should receive more aggressive treatment. Thus, we combined the above biomarkers to build a nomogram to divide patients into two different risk subgroups and explored the treatment value of different CCDs between the subgroups. We paid attention to not only clinical stage and pretreatment EBV DNA level but also post‐IC tumor volume and EBV DNA level as treatment response to IC. The nomogram successfully predicted patients' PFS probabilities and assessed the effect of different CCDs on survival. Grouping patients based on the nomogram, we observed the substantial benefits for both PFS and DMFS from an increasing CCD in the high‐risk subgroup, while no benefits were observed in low‐risk subgroup. In fact, routine decision‐making requires a comprehensive evaluation of not only treatment efficiency, but also patients' tolerance, quality of life, financial situation, and so on. Regardless, the nomogram has potential to aid in decision making.

Notably, the cutoff value of pre‐EBV DNA in our study was different from that used in other studies,^29^, ^30^ which used 2000, 1500, and 4000 copies/ml as the cutoff values. We defined cutoff value according to our data by a ROC curve, which would be a reasonable method. In the current study, the cutoff value of 7000 copies/ml could successfully reflect intrinsic relationship of pre‐EBV DNA and survival outcome. Although the cutoff value of pre‐EBV DNA was distinct among different studies, the results suggested that pretreatment EBV DNA level could serve as an effective marker in predicting survival prognosis and guiding therapeutic decision. However, it should be noted that common laboratory quantitative method could be considered to reduce variability in plasma EBV DNA levels before our cutoff value could be applied widely in clinical practice.

Overall, our findings support the recommendation of a CCD > 200 mg/m^2^ for high‐risk patients. This result could possibly be explained. High‐risk patients had a greater tumor burden and less sensitivity to IC, which may contribute to the higher risk of disease progression. Some of these patients may have subclinical micrometastases at the initial diagnosis and may not benefit significantly from IC, thus, they may need intensified therapy to further reduce the risk of treatment failure. Concurrent chemotherapy can not only play a role in radiotherapy sensitization but also contribute to the eradication of tumor micrometastasis. Therefore, an increasing CCD might provide effective value for longer survival in these patients. However, the low‐risk subgroup had relatively satisfactory survival outcomes with a low CCD. Meanwhile, chemotherapy‐related acute toxicities such as hepatoxicity, hematological toxicities, and gastrointestinal reactions may influence the implementation of radiotherapy. Discontinuing or prolonging treatment can reduce therapeutic efficacy.^31^, ^32^ Therefore, intensified treatment may be needed for high‐risk patients if they can tolerate such therapy according to thorough assessment during therapy, and timely screening of low‐risk patients should be implemented for deintensification of their treatment. This nomogram has good application potential for clinical practice. Doctors could use our nomogram to assess the degree of risk in LA‐NPC patients after IC and decide whether to conduct a high CCD. However, we cannot ignore the fact that some patients at a high‐risk level still experienced treatment failure after receiving a high CCD, indicating that other adjuvant therapies, such as immunotherapy and targeted therapy, could also be taken into consideration for this subgroup to improve the treatment efficiency and finally increase survival rates on the basis of a high CCD.^33^, ^34^, ^35^, ^36^, ^37^

The current study has some limitations. First, although we conducted PSM and multivariate analyses to minimize bias, inherent selective bias was unavoidable because of the retrospective nature of the study. Further prospective trials are needed to validate the conclusion. Second, the study patients were a relatively heterogeneous group with advanced stage who received IC plus CCRT. Hence, the sample size may not be adequate. We expect a larger study population in the future if possible.

In conclusion, this study demonstrated that high‐ and low‐risk groups identified by the nomogram benefit differently from a CCD > 200 mg/m^2^ and thus deserve different treatment strategies. An increasing CCD would improve the treatment efficacy for high‐risk patients but not for low‐risk patients.

## CONFLICT OF INTEREST

None declared.

## AUTHOR CONTRIBUTIONS

Study conception and design: Xiao‐Dong Zhu and Yu‐Ting Jiang. Data acquisition and quality control: all authors; Statistical analysis: Yu‐Ting Jiang and Kai‐Hua Chen; Manuscript preparation: Yu‐Ting Jiang; Manuscript review: all authors.

## ETHICAL APPROVAL STATEMENT

The study was approved by the Medical Ethics Committee of Guangxi Medical University Cancer Hospital.

## Supporting information

Supplementary Material

Supplementary Material

Supplementary Material

Supplementary Material

Supplementary Material

## Data Availability

Data of this study are not publicly available, but data can be made available upon reason‐able request.
