# Supplementary figures and images for: Efficiency of high cumulative cisplatin dose in high‐ and low‐risk patients with locoregionally advanced nasopharyngeal carcinoma
Source: Cancer Med. 2021 Dec 3;11(3):715–27. doi: 10.1002/cam4.4477 (PMC8817101; doi:10.1002/cam4.4477)

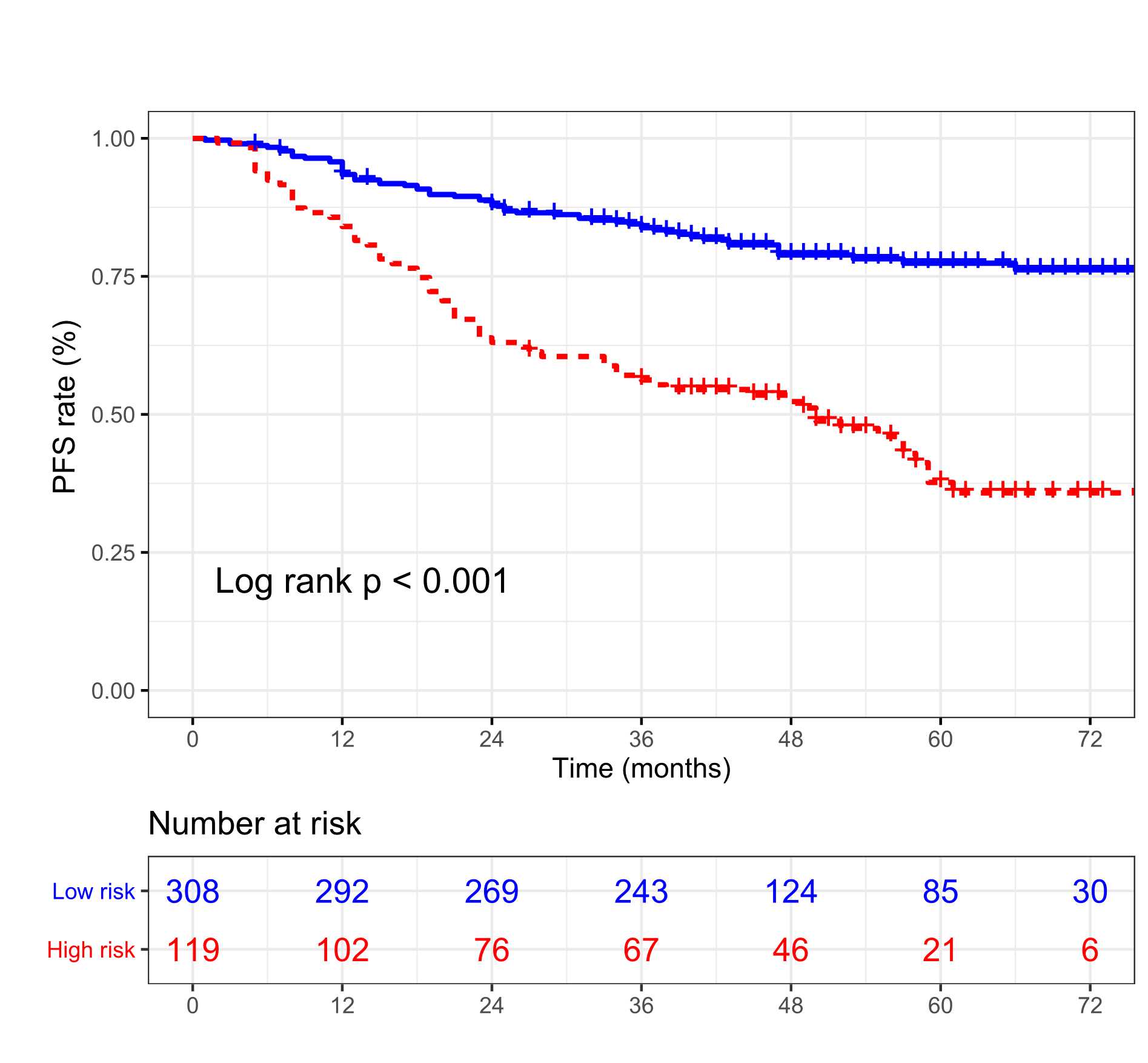

Supplement: Supplementary file 1 — Supplementary Material [file CAM4-11-715-s002.Tiff]

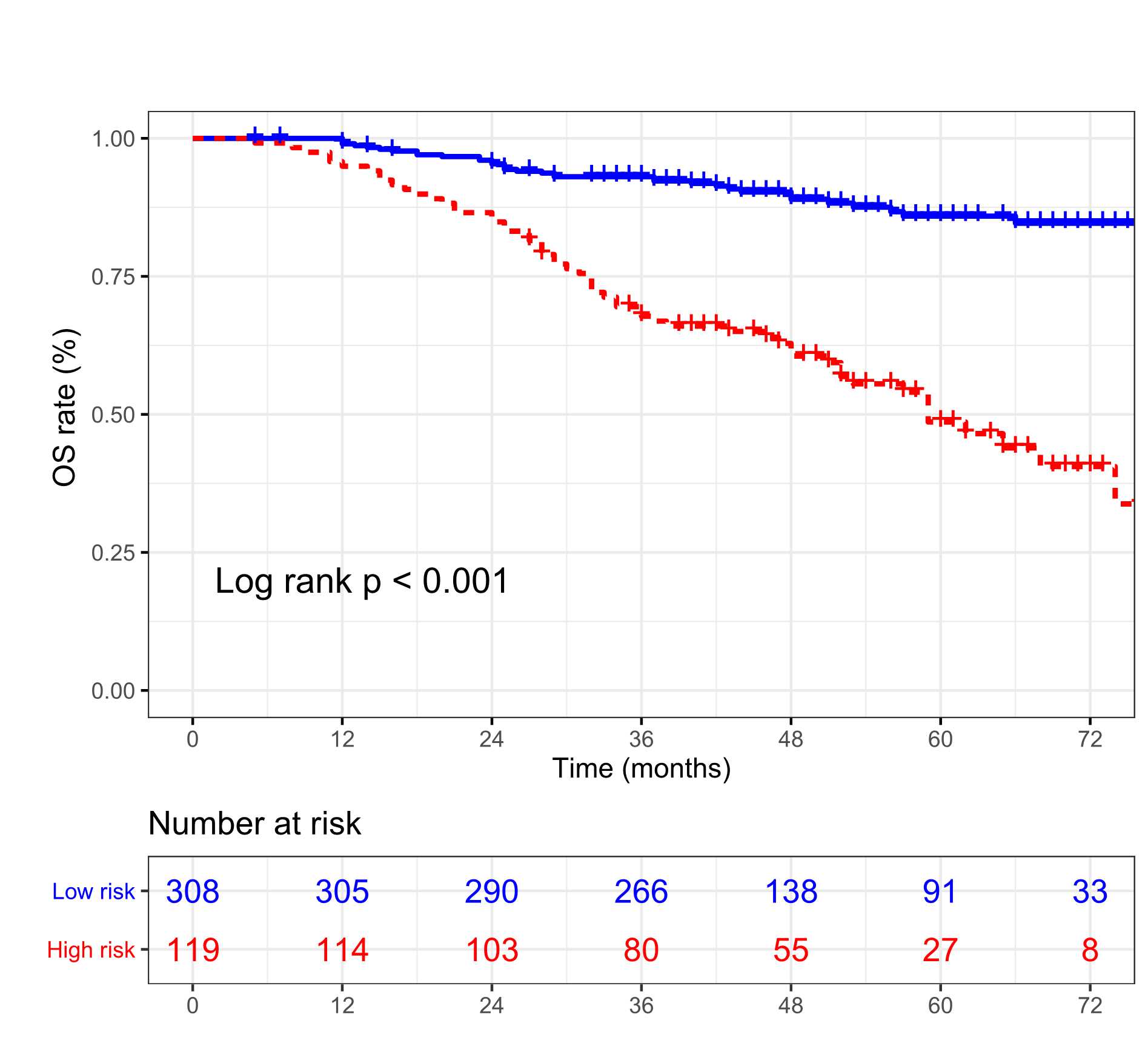

Supplement: Supplementary file 2 — Supplementary Material [file CAM4-11-715-s005.Tiff]

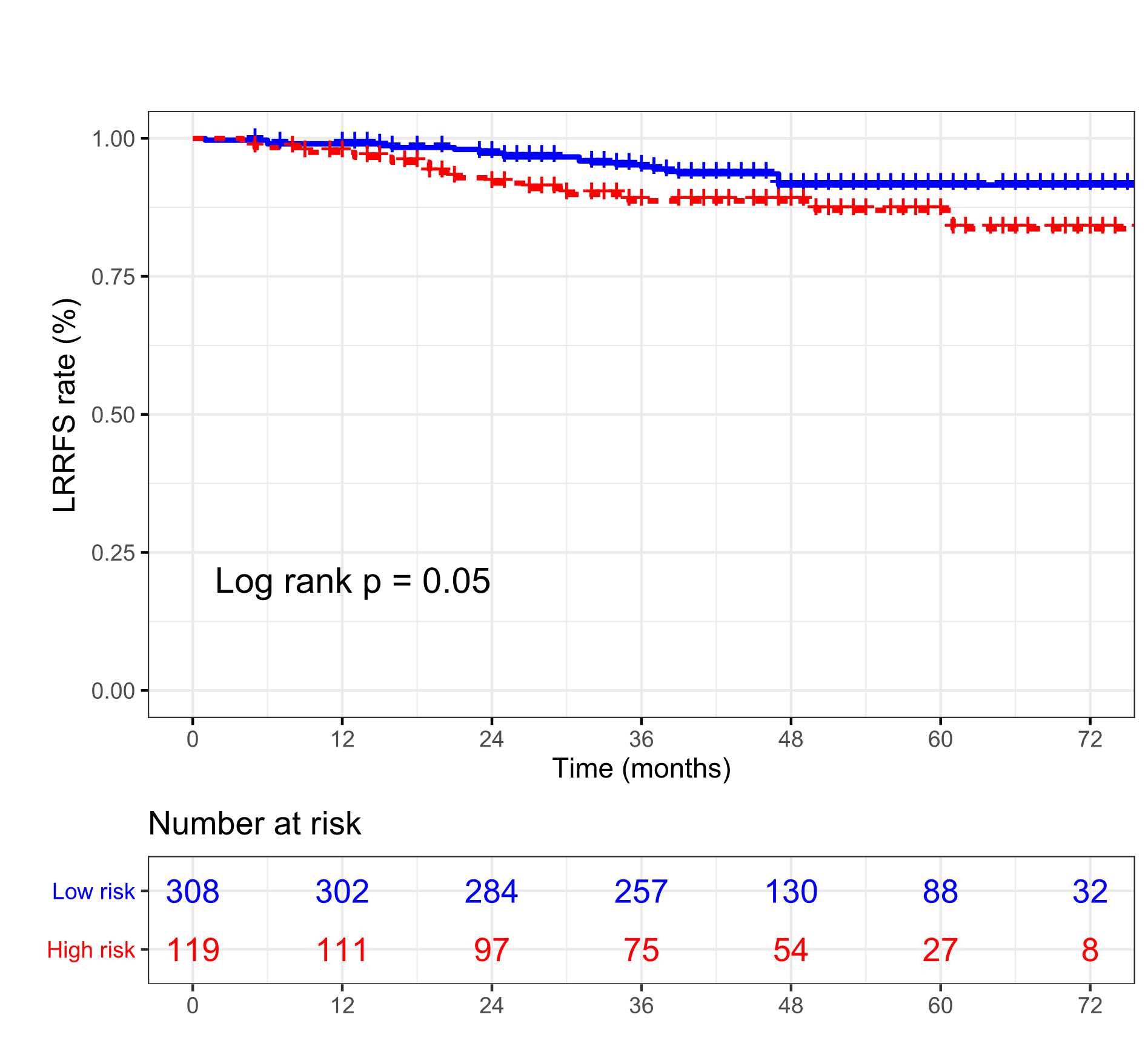

Supplement: Supplementary file 3 — Supplementary Material [file CAM4-11-715-s003.Tiff]

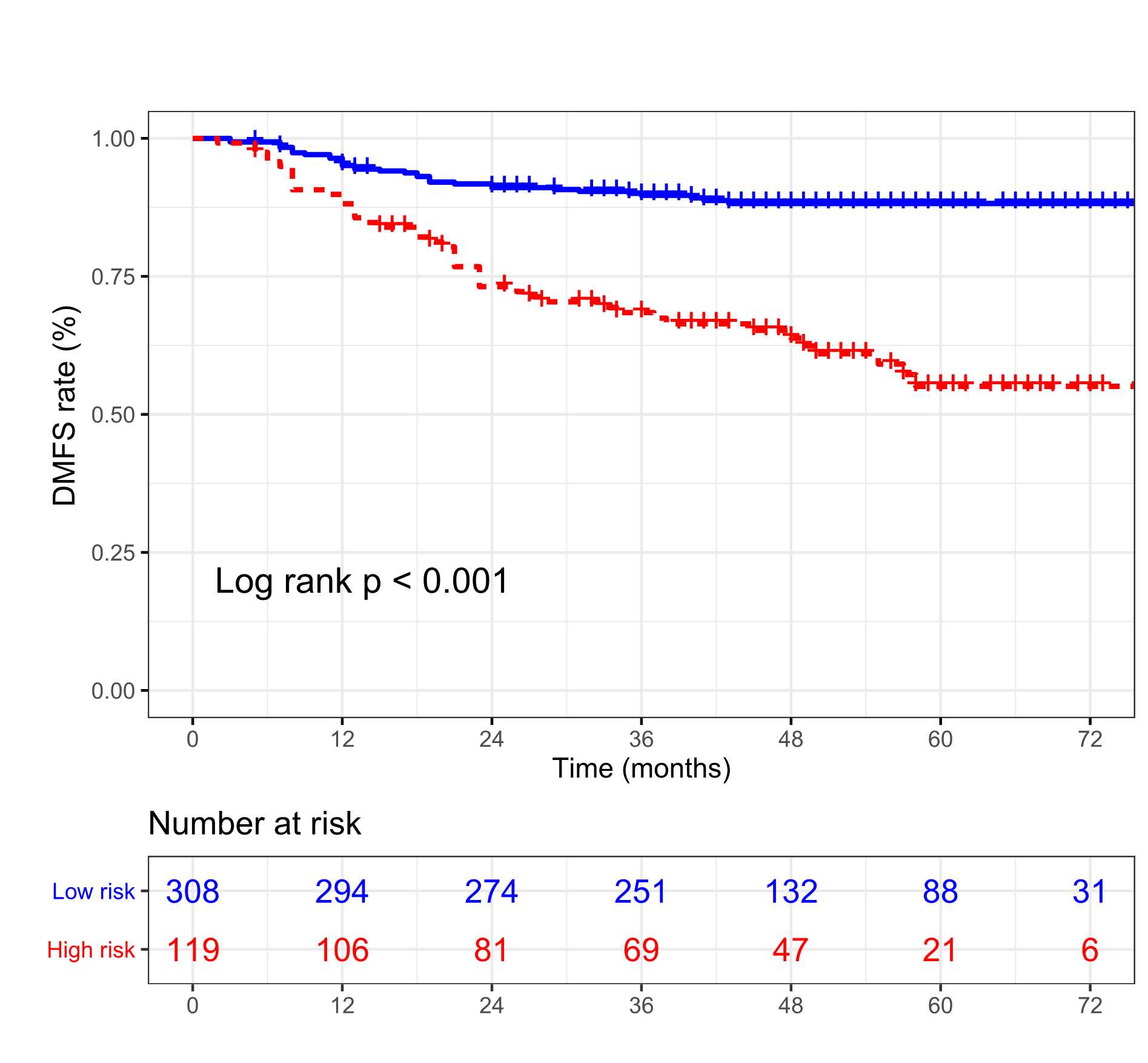

Supplement: Supplementary file 4 — Supplementary Material [file CAM4-11-715-s004.Tiff]
