# Supplementary material for: Efficiency of high cumulative cisplatin dose in high‐ and low‐risk patients with locoregionally advanced nasopharyngeal carcinoma
Source: Cancer Med. 2021 Dec 3;11(3):715–27. doi: 10.1002/cam4.4477 (PMC8817101; doi:10.1002/cam4.4477)
Supplement: Supplementary file 5 — Supplementary Material [file CAM4-11-715-s001.docx]

**Supplementary Table 1.** Univariate Cox regression analysis in the entire cohort.

| Characteristics | HR ( 95%CI) | p-value |
| --- | --- | --- |
| Overall survival |  |  |
| Age (y) (≥ 50 vs. < 50) | 1.530 (1.013-2.309) | 0.043 |
| Sex (Male vs. Female) | 1.834 (1.069-3.147) | 0.028 |
| Pathological type (WHO III vs. WHO I/II) | 0.799 (0.435-1.467) | 0.469 |
| T stage (T3/4 vs. T1/2) | 0.918 (0.579-1.453) | 0.714 |
| N stage (N2/3 vs. N0/1) | 2.131 (1.295-3.507) | 0.003 |
| Clinical stage (IVa vs. III) | 2.600 (1.584-4.267) | <0.001 |
| Post-GTVnx (≥ 118 vs. < 118) | 2.233 (1.471-3.389) | <0.001 |
| Post-GTVnd (≥ 37 vs. < 37) | 2.646 (1.707-4.101) | <0.001 |
| Pre-EBV DNA (≥ 7000 vs. < 7000) | 2.467 (1.631-3.732) | <0.001 |
| Post-EBV DNA (detectable vs. Undetectable) | 2.256 (1.494-3.407) | <0.001 |
| IC regimen (TPF vs. Others) | 0.762 (0.406-1.431) | 0.398 |
| IC cycles (3/4 vs. 2) | 0.965 (0.569-1.635) | 0.894 |
| CCD (> 200 vs. ≤ 200) | 0.822 (0.513-1.318) | 0.416 |
| Progression-free survival |  |  |
| Age (y) (≥ 50 vs. < 50) | 1.077 (0.755-1.535) | 0.682 |
| Sex (Male vs. Female) | 1.261 (0.834-1.906) | 0.271 |
| Pathological type (WHO III vs. WHO I/II) | 1.140 (0.629-2.066) | 0.666 |
| T stage (T3/4 vs. T1/2) | 0.881 (0.602-1.290) | 0.516 |
| N stage (N2/3 vs. N0/1) | 1.909 (1.267-2.875) | 0.002 |
| Clinical stage (IVa vs. III) | 1.997 (1.355-2.943) | <0.001 |
| Post-GTVnx (≥ 118 vs. < 118) | 1.633 (1.133-2.353) | 0.009 |
| Post-GTVnd (≥ 37 vs. < 37) | 2.158 (1.509-3.085) | <0.001 |
| Pre-EBV DNA (≥ 7000 vs. < 7000) | 2.731 (1.752-4.258) | <0.001 |
| Post-EBV DNA (detectable vs. Undetectable) | 2.189 (1.408-3.402) | <0.001 |
| IC regimen (TPF vs. Others) | 0.763 (0.452-1.288) | 0.311 |
| IC cycles (3/4 vs. 2) | 0.988 (0.630-1.551) | 0.959 |
| CCD (> 200 vs. ≤200) | 0.683 (0.452-1.032) | 0.070 |
| Locoregional relapse-free survival |  |  |
| Age (y) (≥ 50 vs. < 50) | 0.946 (0.471-1.902) | 0.877 |
| Sex (Male vs. Female) | 1.070 (0.501-2.283) | 0.826 |
| Pathological type (WHO III vs. WHO I/II) | 1.873 (0.449-7.808) | 0.389 |
| T stage (T3/4 vs. T1/2) | 0.875 (0.420-1.823) | 0.722 |
| N stage (N2/3 vs. N0/1) | 0.926 (0.465-1.841) | 0.826 |
| Clinical stage (IVa vs. III) | 1.383 (0.688-2.780) | 0.362 |
| Post-GTVnx (≥ 118 vs. < 118) | 1.946 (0.980-3.865) | 0.057 |
| Post-GTVnd (≥ 37 vs. < 37) | 1.597 (0.816-3.123) | 0.172 |
| Pre-EBV DNA (≥ 7000 vs. < 7000) | 1.660 (0.851-3.236) | 0.137 |
| Post-EBV DNA (detectable vs. Undetectable) | 1.256 (0.622-2.536) | 0.524 |
| IC regimen (TPF vs. Others) | 0.505 (0.155-1.651) | 0.259 |
| IC cycles (3/4 vs. 2) | 1.277 (0.495-3.292) | 0.613 |
| CCD (> 200 vs. ≤ 200) | 0.836 (0.391-1.784) | 0.642 |
| Distant metastasis-free survival |  |  |
| Age (y) (≥ 50 vs. < 50) | 0.908 (0.571-1.444) | 0.684 |
| Sex (Male vs. Female) | 1.138 (0.680-1.904) | 0.623 |
| Pathological type (WHO III vs. WHO I/II) | 0.984 (0.473-2.043) | 0.965 |
| T stage (T3/4 vs. T1/2) | 0.728 (0.456-1.162) | 0.183 |
| N stage (N2/3 vs. N0/1) | 3.123 (1.688-5.776) | <0.001 |
| Clinical stage (IVa vs. III) | 1.795 (1.106-2.914) | 0.018 |
| Post-GTVnx (≥ 118 vs. <118) | 1.277 (0.787-2.073) | 0.322 |
| Post-GTVnd (≥ 37 vs. < 37) | 3.227 (1.973-5.277) | <0.001 |
| Pre-EBV DNA (≥ 7000 vs. < 7000) | 2.731 (1.752-4.258) | <0.001 |
| Post-EBV DNA (detectable vs. Undetectable) | 2.189 (1.408-3.402) | <0.001 |
| IC regimen (TPF vs. Others) | 0.710 (0.355-1.420) | 0.333 |
| IC cycles (3/4 vs. 2) | 1.185 (0.641-2.189) | 0.588 |
| CCD (> 200 vs. ≤ 200) | 0.657 (0.384-1.122) | 0.124 |

Abbreviations: CI, confidence interval; HR, hazard ratio; WHO, World Health Organization; IC, induction chemotherapy; CCD, cumulative cisplatin dose; EBV, Epstein–Barr virus; pre-EBV DNA, pretreatment EBV DNA; post-EBV DNA, post-IC EBV DNA; post-GTVnx, post-IC primary gross tumor volume; post-GTVnd, post-IC cervical lymph node tumor volume; cc, cubic centimeter.

**Supplementary Table 2** Multivariable Cox regression analysis in the entire cohort.

| Characteristics | HR (95%CI) | p-value |
| --- | --- | --- |
| Overall survival |  |  |
| Age (y) (≥ 50 vs. < 50) | 1.482 (0.975-2.255) | 0.066 |
| Sex (Male vs. Female) | 1.538 (0.892-2.650) | 0.121 |
| Clinical stage (IVa vs. III) | 1.882 (1.120-3.162) | 0.017 |
| Post-GTVnx (≥ 118 vs. < 118) | 1.574 (1.012-2.451) | 0.044 |
| Post-GTVnd (≥ 37 vs. < 37) | 2.404 (1.545-3.741) | <0.001 |
| Post-EBV DNA (detectable vs. Undetectable) | 1.783 (1.163-2.734) | 0.008 |
|  |  |  |
| Progression-free survival |  |  |
| Clinical stage (IVa vs. III) | 1.643 (1.097-2.462) | 0.016 |
| Post-GTVnx (≥ 118 vs. < 118) | 1.315 (0.900-1.922) | 0.157 |
| Post-GTVnd (≥ 37 vs. < 37) | 2.029 (1.417-2.905) | <0.001 |
| Post-EBV DNA (detectable vs. Undetectable) | 1.653 (1.156-2.363) | 0.006 |
|  |  |  |
| Distant metastasis-free survival |  |  |
| Clinical stage (IVa vs. III) | 1.746 (1.178-2.589) | 0.006 |
| Post-GTVnd (≥ 37 vs. < 37) | 2.014 (1.406-2.884) | <0.001 |
| Post-EBV DNA (detectable vs. Undetectable) | 1.702 (1.194-2.426) | 0.003 |

Abbreviations: CI, confidence interval; HR, hazard ratio; EBV, Epstein–Barr virus; post-EBV DNA, post-IC EBV DNA; post-GTVnx, post-IC primary gross tumor volume; post-GTVnd, post-IC cervical lymph node tumor volume.

A Cox proportional hazards regression model was used to detect variables individually without adjustment. All variables were transformed into categorical variables. HRs were calculated for age (years) (≥ 50 vs. < 50), sex (male vs. female), clinical stage (IVa vs. III), post-EBV DNA (detectable vs. Undetectable), post-GTVnx (≥ 118 cc vs. <118 cc), post-GTVnx (≥ 37 cc vs. <37 cc).

**Supplementary Figure 1** PFS (A), OS (B), LRRFS (C) and DMFS (D) Kaplan–Meier curves for nomogram-defined low- and high-risk subgroups.

**Supplementary materials:**

**Details of chemotherapy and radiotherapy treatment**

The regimens of IC included TPF regimen (docetaxel with cisplatin with 5-fluorouracil, 60, 60, and 3,000 mg/m2, respectively), or TP regimen (docetaxel with cisplatin, 75 and 75 mg/m2, respectively), or GP regimen (gemcitabine with cisplatin, 1000 and 80 mg/m2, respectively), or PF regimen (cisplatin with 5-fluorouracil, 80 and 4,000 mg/m2, respectively).

The primary gross tumor volume (GTVnx) and cervical lymph node tumor volume (GTVnd) included the entire macroscopic tumor defined with the aid of computed tomography (CT), magnetic resonance imaging (MRI) scans, and physical examinations. Two clinical target volumes (CTVs) were delineated according to the tumor invasion pattern. The high-risk clinical target volume (CTV1) included the GTVnx add a margin of 0.5 to 1 cm (forward, both sides, up and down) and a margin of 0.3 to 0.5 cm (back) to encompass the high-risk sites of microscopic extension and the whole nasopharynx. The low-risk clinical target volume (CTV2) was defined as the CTV1 add a margin of 0.5 to 1 cm (forward, both sides, up and down) and a margin of 0.3 to 0.5 cm (back) to encompass the low-risk sites of microscopic extension, the GTVnd, and elective neck area from level IB to V.

**Details of measurements of EBV DNA level:**

The pretreatment EBV DNA level (pre-EBV DNA) and EBV DNA level after IC (post-EBV DNA) were measured by real-time quantitative polymerase chain reaction (PCR) technique amplifying the BamHI-W fragment region of the EBV genome before and after IC. The results are shown as the number of copies of the EBV genome per milliliter of plasma.

**Details of measurements of post-IC tumor volumes:**

All NPC patients were immobilized with a tailor-made thermoplastic cast from head to shoulders. Contrast-enhanced computed tomography (CT) simulation scans at the radiotherapy position were performed on all patients. The scope of each scan was performed with a thickness of 3 mm from the top of the head to 2 cm below the lower edge of the clavicle. The contrastenhanced CT images were transmitted into the radiotherapy planning system. The post-IC GTVnx and GTVnd were delineated on each slice of planning CT images according to the post-IC MRI image and calculated automatically by the treatment planning system. Retropharyngeal lymph nodes were encompassed in the GTVnx, as the retropharyngeal lymph node and primary tumor are so close that the discrimination of these anatomical sites remains difficult. The GTVnd included metastatic cervical lymph nodes and nodes with necrosis or extracapsular spread and nodal extracapsular spread based on pretreatment MRI.
